# Surface-Modified Graphene Oxide/Lead Sulfide Hybrid Film-Forming Ink for High-Efficiency Bulk Nano-Heterojunction Colloidal Quantum Dot Solar Cells

**DOI:** 10.1007/s40820-020-00448-8

**Published:** 2020-05-16

**Authors:** Yaohong Zhang, Guohua Wu, Chao Ding, Feng Liu, Dong Liu, Taizo Masuda, Kenji Yoshino, Shuzi Hayase, Ruixiang Wang, Qing Shen

**Affiliations:** 1 Faculty of Informatics and Engineering, The University of Electro-Communications, Tokyo, 182-8585 Japan; 2 School of Materials Science and Engineering, Shaanxi Normal University, Xi’an, 710119 People’s Republic of China; 3 X-Frontier Division, Toyota Motor Corporation, Shizuoka, 471-8571 Japan; 4 Department of Electrical and Electronic Engineering, University of Miyazaki, Miyazaki, 889-2192 Japan; 5 Beijing Engineering Research Centre of Sustainable Energy and Buildings, Beijing University of Civil, Engineering and Architecture, Beijing, 102616 People’s Republic of China

**Keywords:** Quantum dot solar cells, PbS colloidal quantum dots, Hole extraction, Graphene oxide, Surface modified

## Abstract

Solution-processed colloidal quantum dot solar cells (CQDSCs) is a promising candidate for new generation solar cells. To obtain stable and high performance lead sulfide (PbS)-based CQDSCs, high carrier mobility and low non-radiative recombination center density in the PbS CQDs active layer are required. In order to effectively improve the carrier mobility in PbS CQDs layer of CQDSCs, butylamine (BTA)-modified graphene oxide (BTA@GO) is first utilized in PbS-PbX_2_ (X = I^−^, Br^−^) CQDs ink to deposit the active layer of CQDSCs through one-step spin-coating method. Such surface treatment of GO dramatically upholds the intrinsic superior hole transfer peculiarity of GO and attenuates the hydrophilicity of GO in order to allow for its good dispersibility in ink solvent. The introduction of BTA@GO in CQDs layer can build up a bulk nano-heterojunction architecture, which provides a smooth charge carrier transport channel in turn improves the carrier mobility and conductivity, extends the carriers lifetime and reduces the trap density of PbS-PbX_2_ CQDs film. Finally, the BTA@GO/PbS-PbX_2_ hybrid CQDs film-based relatively large-area (0.35 cm^2^) CQDSCs shows a champion power conversion efficiency of 11.7% which is increased by 23.1% compared with the control device.

**Electronic supplementary material:**

The online version of this article (10.1007/s40820-020-00448-8) contains supplementary material, which is available to authorized users.

## Highlights

- Butylamine-modified graphene oxide (BTA@GO) is first utilized in PbS colloidal quantum dots (CQDs) ink to deposit the active layer of colloidal quantum dot solar cells (CQDSCs).
- The BTA@GO improves the carrier transfer rate in the CQDs active layer and 11.7% conversion efficiency is achieved.

**Electronic supplementary material:**

The online version of this article (10.1007/s40820-020-00448-8) contains supplementary material, which is available to authorized users.

## Graphical abstract

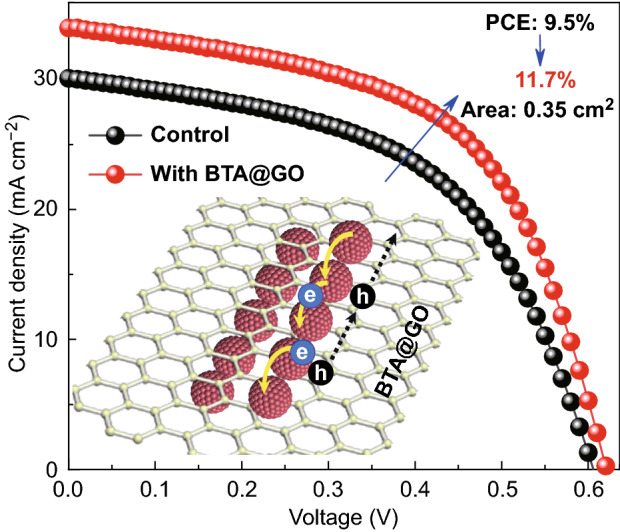

## Introduction

Semiconductor colloidal quantum dots (CQDs) are attracting enormous attention in the next-generation photovoltaic applications due to their size-dependent bandgap tunability, facile solution-processed manufacturability and especially multiple exciton generation (MEG) effect [1, 2]. The photovoltaic performance of colloidal quantum dot solar cells (CQDSCs) has been significantly improved in the past few years by CQDs surface ligand modification [3–9], device architecture optimization [10–15], active layer deposition engineering [16–18], etc. Nevertheless, the performance of CQDSCs is still much lower than its theoretical efficiency (44.4%) [19, 20]. Recently, a certified record power conversion efficiency (PCE) of 16.6% has been achieved by Cs_0.5_FA_0.5_PbI_3_ perovskite-based CQDSCs [19], and the PCE of lead sulfide (PbS)-based CQDSCs reached 13.3% with aperture area of 0.049 cm^2^ [21]. There are many challenges and issues waiting to be solved on the way forward to further improve the performance of CQDSCs.

Photoexcited carrier diffusion length and recombination centers are crucial for CQDs active layer because they closely relate to the charge separation and collection efficiencies and short-circuit photocurrent density (*J*_SC_) as well as open-circuit photovoltage (*V*_OC_) of CQDSCs devices. The PCE of CQDSCs is also limited by the common trade-off between light absorption and charge carrier extraction in CQDs layer. To further balance the charge extraction efficiencies and light absorption, n-type semiconductor (i.e., ZnO) nanowire electrode with large electron diffusion coefficient has been introduced into CQDSCs to build a bulk heterojunction architecture in favor of improving the charge separation at the electrode/CQDs interface and electron diffusion length, which results in possible increase of the thickness of CQDs active layer [18, 22–25]. Although the use of nanowire electrode can improve the *J*_SC_ of the device to a certain extent due to the increased thickness of CQDs active layer, the *V*_OC_ and fill factor (FF) of these devices are usually deteriorated compared to the planar heterojunction CQDSCs. The reasons for this phenomenon can be summed up in three ways: (1) numerous defect states in nanowire electrode lead to great charge trapping; (2) the enhanced interfacial charge carrier recombination through the large interface area between the n-type nanowire electrode and CQDs; (3) the smaller hole diffusion length or extraction rate in CQDs layer enhances the charge recombination which offsets the contribution of photogenerated charge carriers to *J*_SC_. What needs to pay special attention is that the value of hole mobility of PbS CQD has been found to be much smaller than its electron mobility [26]. How to effectively improve the hole mobility of PbS CQDs active layer to reduce recombination and improve hole collection efficiency in PbS CQDs layer is an urgent issue for CQDSCs. This issue settlement will be beneficial to elongate the carrier lifetime and fully harnesses great potential for the advance in the photovoltaic performance of CQDSCs. Unfortunately, scarcely related work has been reported nowadays.

Graphene oxide (GO) has been successfully employed as hole transport layer to improve the hole extraction rate from active layer to counter electrode in many typical solar cells such as polymer solar cells [27], perovskite solar cells [28], and CQDSCs due to its superior hole extraction efficiency [29–31]. It is very interesting that the surface of GO can also be chemically modified by special organic materials. Surface-modified GO can uphold the intrinsic hole transfer peculiarity of GO on the one hand and attenuate the hydrophilicity of GO in order to possibly allow for its good dispersibility in organic solvents on the other hand. Taking the well dispersed solvent of CQD into consideration, butylamine (BTA) is a good option to chemically modify the surface of GO.

Unlike the utilization of GO as a hole transport layer, we herein introduce the surface-modified GO using BTA by exploiting the bonding between the functional groups on the surface of GO and BTA to prepare BTA@GO/PbS-PbX_2_ hybrid CQDs active layer in order to elevate the inherent hole mobility of CQDs layer. Here we deposit the CQDs active layer of PbS CQDSCs via one-step deposition strategy using BTA@GO/PbS-PbX_2_ hybrid CQDs ink. It is found that the introduction of BTA@GO can build up a bulk nano-heterojunction architecture, which effectively improves the charge transfer rate and carrier mobility, extends the carriers lifetime and reduces the trap density of PbS-PbX_2_ CQDs film. The BTA@GO/PbS-PbX_2_ hybrid CQDs film-based relatively large-area (0.35 cm^2^) CQDSCs shows a champion PCE of 11.7% with *J*_SC_ of 33.9 mA cm^−2^, *V*_OC_ of 0.622 V and FF of 0.555. Compared to the PCE of 9.5% achieved by the PbS-PbX_2_ CQDs film-based control device, the efficiency is increased by 23.1%. In addition, the BTA@GO/PbS-PbX_2_ hybrid CQDs film-based device exhibits good output and air storage stability.

## Experimental

### Materials

Oleic acid (OA, 90%), 1-octadecene (ODE, 90%), oleylamine (OLA, 70%), tetradecylphosphonic acid (TDPA, 97%), 1,2-ethanedithiol (EDT, 98%), hexamethyldisilathiane (TMS), lead bromide (PbBr_2_, 95%), and poly(3,4-ethylenedioxythiophene)-poly(styrenesulfonate) (PEDOT:PSS) were purchased from Sigma-Aldrich. Lead (II) oxide (PbO, 99.5%), cadmium chloride (CdCl_2_, 95%), ammonium acetate (NH_4_Ac, 95%), *N,N*-dimethylformamide (DMF, 99.7%), octane (99%), butylamine (BTA, 99%), acetone (99.7%), methanol (99.7%), and toluene (99%) were purchased from Wako. Lead iodide (PbI_2_, 99%) was purchased from Kanto Chemicals. Graphene oxide (GO, product number: XF002-1) was purchased from Nanjing XFNANO Materials. All chemicals were used without any purification.

### Synthesis of PbS CQD

PbS CQD was synthesized by using our previously reported method [6]. Briefly, 6 mmol of PbO and 15 mmol of OA were dissolved in 50 mL of ODE by degassing the mixture at room temperature and 80 °C under vacuum for 0.5 and 2 h, respectively. Under nitrogen atmosphere, 630 μL TMS together with 10 mL ODE was quickly injected into the lead precursor at 85 °C. When the obtained PbS CQD solution was cooled to 70 °C, CdCl_2_-TDPA-OLA halide passivation solution was injected. After the mixture solution was cooled to room temperature, PbS CQD was purified by acetone/methanol mixture solution. The obtained PbS CQD precipitate was dried by nitrogen flow and diluted in octane (100 mg mL^−1^).

### Preparation of BTA@GO Solution

20 mL BTA containing monolayer GO sheet (0.1–0.3 mg mL^−1^) was sonicated for about 1 h in an ultrasonic bath, and then the mixture was stirred under reflux condition at 60 °C for 24 h. After that, a well-dispersed BTA@GO solution was obtained.

### Preparation of PbS CQDs Ink

The PbS CQDs ink was prepared by a reported solution-phase ligand-exchange method [16, 17]. Briefly, 12 mL OA-capped PbS (PbS-OA) CQD octane solution (20 mg mL^−1^) was dropwise added into 20 mL DMF solution which dissolves 0.1 M PbI_2_, 0.04 M PbBr_2_ and 0.06 M NH_4_Ac with vigorous stirring. This mixture was continued to stir for 5 min after PbS CQD octane solution dropping out. The upper octane phase was removed, and the DMF phase was washed for three times with octane. Then the PbX_2_ (X = I, Br)-capped PbS (PbS-PbX_2_) CQDs were precipitated from DMF by adding 15 mL of toluene and were collected by centrifugation at 7500 rpm for 5 min. The obtained PbS-PbX_2_ CQD precipitate was then dried in a vacuum oven at room temperature for 30 min and dispersed in BTA or BTA@GO solution.

### Photovoltaic Device Fabrication

The FTO/TiO_2_ substrate was prepared following a published method [17]. The substrate was annealed again and further treated with oxygen plasma for 10 min before use. The CQDs active layer was deposited onto FTO/TiO_2_ substrate by a one-step spinning 120 μL CQDs ink at 2500 rpm for 1 min. The obtained FTO/TiO_2_/CQDs film was dried in vacuum oven at room temperature for 3 h. Subsequently, two PbS-EDT CQD layers (about 110 nm) were deposited onto FTO/TiO_2_/CQDs film by a layer-by-layer spin-coating deposition method. Finally, Au top electrode was thermally evaporated onto the PbS-EDT CQDs film. Four identical solar cells are integrated on each substrate with an active area of 0.35 cm^2^.

### Characterization

Fourier transform infrared (FTIR) spectra of GO, BTA and BTA@GO were performed using a Thermo Scientific Nicolet 6700 FTIR spectrometer. Transmission electron microscope (TEM) images of CQDs and GO were determined by JEOL JEM-2100F. Scanning electron microscope (SEM) images were examined using JEOL JSM-6340. Atomic force microscope (AFM) images were measured using SHIMADZU SPM-9700. Photoelectron yield spectroscopy (PYS) was obtained by Bunkoukeiki BIP-KV205. Absorption spectrum was captured by using a JASCO V-670 spectrophotometer. Time-resolved transient absorption (TA) measurement was carried out on a fs-TA system. PbS CQD films for TA measurement were sealed into a transparent quartz cuvette (size: 60 × 10 × 40 mm^3^) filled with N_2_ gas, and the excitation positions of sample were moved during the measurement using a XY translational stage. The microwave photoconductivity decay (*μ*-PCD) measurement was carried out on JEIDA-53 with a UV 349 nm excitation light and a differential *μ*-PCD (26 GHz) detection method. The current density–voltage (*J*–*V*) curve of device was obtained using a Keithley 2400 digital source meter in the dark and under one-sun irradiation (AM 1.5 G 100 mW cm^−2^). The incident photon to current conversion efficiency (IPCE) measurement was recorded by using CEP-2000SRR, Bunko Keiki with a 300 W Xe lamp. Electrochemical impedance spectroscopy (EIS) of solar cell device was obtained by using a SP-300 (BioLogic) impedance analyzer with a bias of 0.55 V (amplitude 10 mV).

## Results and Discussion

### Fabrication of BTA@GO/PbS-PbX_2_ Hybrid CQDs Ink

**Fig. 1:**
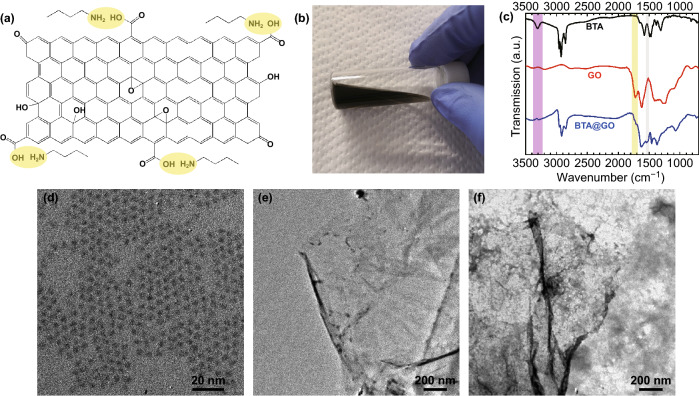
**a** Chemical structure of the GO and the formation mechanism of BTA@GO. **b** Photograph of BTA@GO solution (0.3 mg mL^−1^). **c** FTIR spectra of BTA, GO and BTA@GO on glass/Au substrate. **d** TEM image of colloidal PbS-OA CQD. TEM images of **e** BTA@GO and **f** PbS-PbX_2_ CQD coupled with BTA@GO, the scale bar is 200 nm

In order to effectively disperse the GO sheet without aggregation or precipitation, BTA was first used to chemically modify the surface of the GO sheet and obtain the uniformly dispersed BTA@GO/PbS hybrid CQDs ink. As it is known to all, the surface of GO sheet has many functional groups, such as carboxyl and hydroxyl [32]. Among them, carboxyl group can readily react with the amide unit in the BTA to make a stable BTA@GO solution as illustrated in Fig. 1a. After a brief ultrasonic treatment for the GO and BTA complexes, a stable BTA@GO dispersion is formed. Figure 1b shows the photograph of dispersions of GO in BTA at 0.3 mg mL^−1^ concentration. The chemical changes occurring between GO with BTA can be observed by FTIR in Fig. 1c. For BTA, the peak at 2962 cm^−1^ is assigned to the asymmetric CH_3_ stretch of BTA. The peaks at 2935 and 2871 cm^−1^ which belong to the C–H stretching vibrations of the hydrocarbon chains of the BTA. The strong peak at around 3300 cm^−1^ is assigned to the N–H stretching of BTA. For bare GO, the peak at around 3120–3500 cm^−1^ attributes to the –OH group (H bonded), C=O stretching of carboxyl group at 1715 cm^−1^ and carbonyl group at 1620 cm^−1^, C–OH bond stretch at 1250 cm^−1^, and C–O bond stretching of the epoxy group at from 900 to 1150 cm^−1^ can identify the types of functional groups on the surface of GO. Clearly, in BTA@GO, the presence of three new peaks at 2955, 2918, and 2850 cm^−1^ belongs to C–H stretching vibrations of the hydrocarbon chains of the BTA anchored onto the GO surface. In addition, the significant intensity decrements of the peaks at 1715 and 3300 cm^−1^ and the generation of new peak around 1530 cm^−1^ which is associated with the C–N stretching and N–H bending of secondary amides in BTA@GO confirm the formation of new amide bond in BTA@GO (Fig. S1). TEM images of PbS-OA CQD, BTA@GO, PbS-PbX_2_ CQD, and BTA@GO/PbS-PbX_2_ hybrid CQD are shown in Figs. 1d–f and S2. It is confirmed that the CQD-CQD distance for PbS-PbX_2_ CQD is narrowed after solution-state ligand exchange comparing with PbS-OA CQD.

### Photogenerated Carrier Transfer in BTA@GO/PbS-PbX_2_ Hybrid CQDs Film

**Fig. 2:**
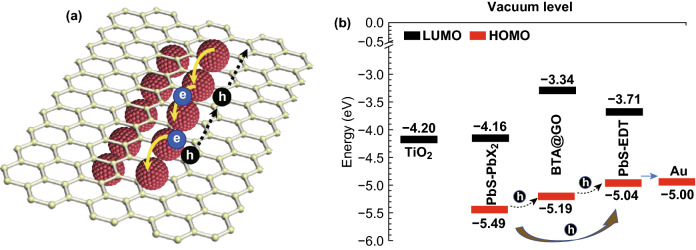
**a** Schematic diagram of the charge transport process in the BTA@GO/PbS-PbX_2_ hybrid CQDs film. **b** Schematic energy level diagram of the TiO_2_, PbS-PbX_2_ CQDs, BTA@GO, PbS-EDT CQDs, and Au

To clearly investigate the energy level matching degree between the surface-modified GO named BTA@GO and PbS-PbX_2_ CQDs or PbS-EDT CQDs, the highest occupied molecular orbital (HOMO) positions of PbS-PbX_2_ CQDs and BTA@GO are measured by using PYS. We find that the color of GO films is different when they are deposited in different solvents. The GO film deposited by using H_2_O@GO solution appears a brown color (Fig. S3a) which is the same as the color of original GO powder, and the film deposited by using BTA@GO solution shows a dark brown color (Fig. S3b). It is mainly caused by the changing of the functional groups on the GO surface [33, 34]. After BTA chemical treatment, the original carboxyl groups on GO can be converted to new amides which has been confirmed by FTIR (Figs. 1c and S1). This change affects not only the color of GO but also its HOMO level. The values of HOMO positions of H_2_O@GO and BTA@GO are − 5.22 and − 5.19 eV, respectively (Fig. S3a, b). The values of HOMO positions of PbS-PbX_2_ CQDs and PbS-EDT CQDs are − 5.49 and − 5.04 eV, respectively (Fig. S3c, d). It means that the photogenerated holes in PbS-PbX_2_ CQDs layer which contract with or near BTA@GO tend to inject from PbS-PbX_2_ CQDs to BTA@GO, and eventually fast transported through GO to PbS-EDT CQDs (Fig. 2b). In contrast, the photogenerated holes in PbS-PbX_2_ CQDs layer which are far from BTA@GO should move to the interface of PbS-PbX_2_/PbS-EDT layers via slow diffusion in PbS-PbX_2_ CQDs layer. The optical bandgap of BTA@GO is approximately 1.85 eV (Fig. S3e), and the calculated LUMO level of BTA@GO is − 3.34 eV which is much higher than the LUMO position of PbS-PbX_2_ CQDs. This high-energy barrier can suppress the photogenerated electrons inject from PbS-PbX_2_ CQDs to BTA@GO. The electrons in PbS-PbX_2_ CQDs layer tend to move to the electron collection layer (TiO_2_) under the driving force of the build-in electric field in the solar cell device. This consideration is then substantiated by measuring the photoexcited charge carrier transfer for PbS-PbX_2_ and BTA@GO/PbS-PbX_2_ CQD solid films using time-resolved TA spectroscopy.

**Fig. 3:**
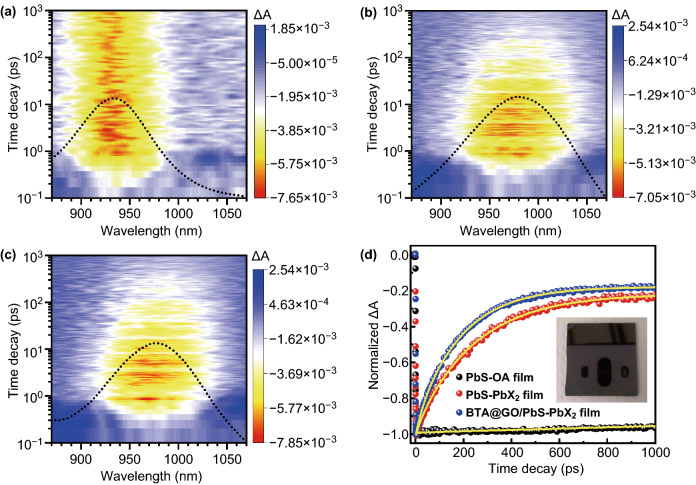
Comparison of the TA decays of PbS-OA CQDs film, PbS-PbX_2_ CQDs film and BTA@GO/PbS-PbX_2_ hybrid CQDs film. **a-c** TA and ground-state absorption spectra for PbS-OA, PbS-PbX_2_ and BTA@GO/PbS-PbX_2_ CQD films. The excitation wavelength is 470 nm with a pulse fluence of 20 μJ cm^−2^. **d** Normalized absorption changes (△A) of those films; the films are excited by 470 nm pulse with a fluence of 6 μJ cm^−2^. The inset is the photograph of BTA@GO/PbS-PbX_2_ CQDs film, and the shadow of the phone used to take photo is reflected in the mirror-like surface of the CQDs film

TA and ground-state absorption spectra of PbS-OA, PbS-PbX_2_, and BTA@GO/PbS-PbX_2_ CQD films are shown in Figs. 3a–c and S4, respectively. It can be manifestly observed that both TA bleaching peak and ground-state absorption peak around 930 nm (1.33 eV) of the PbS-OA CQDs correspond to the lowest-energy excitons in the CQDs. For the CQD film prepared using the CQD ink after solution-phase ligand exchange, due to the narrowed CQD-CQD distance and the enhanced coupling effect between PbS CQDs in the film, the TA bleaching peaks and optical absorption peaks for both of PbS-PbX_2_ CQDs and BTA@GO/PbS-PbX_2_ CQDs are slightly redshifted to 970 nm. In order to eliminate the interference of Auger recombination and obtain large signal-to-noise ratio, a weak excitation fluence of 6 μJ cm^−2^ is adopted in the TA measurement (Fig. S5). In Figs. 3d and S6, compared with PbS-OA CQDs (probe wavelength 930 nm), obviously fast TA bleaching signal decay (probe wavelength 970 nm) for both PbS-PbX_2_ CQDs and BTA@GO/PbS-PbX_2_ CQDs films can be observed on the time scale of 1 ns. The fast TA bleaching signal decay indicates charge carriers transfer occurring in those CQD films. The TA decay curves can be well fitted by biexponential decay with a constant signal, and the fitting results are shown in Table 1. The faster decay is contributed to charge carrier trapping of PbS CQDs (parameters are represented as *τ*_tr_). The relatively slower decay process corresponds to the charge carrier transfer behavior between CQD-CQD and CQD-BTA@GO (parameters are represented as *τ*_ct_). The values of carrier transfer time constant *τ*_ct_ for PbS-PbX_2_ CQDs film and BTA@GO/PbS-PbX_2_ hybrid CQDs film are about 243 ± 4 and 180 ± 3 ps, respectively. Also, the corresponding carrier transfer rate constants (*k*_ct_ = 1/*τ*_ct_) of PbS-PbX_2_ CQDs film and BTA@GO/PbS-PbX_2_ hybrid CQDs film are 4.1 × 10^9^ and 5.6 × 10^9^ s^−1^, respectively. It means that charge carrier transfer between CQDs in the film prepared using the BTA@GO/PbS-PbX_2_ hybrid CQDs ink is faster than that in the film deposited using the PbS-PbX_2_ CQDs ink due to the contribution of the charge carrier transfer process between CQD and BTA@GO. This result reflects that PbS-PbX_2_ CQDs can closely couple with BTA@GO in the BTA@GO/PbS-PbX_2_ hybrid CQDs film. The carrier transfer rate from PbS-PbX_2_ CQD to BTA@GO can be estimated by Eq. 1:

1 $$k_{{{\text{CQD-BTA@GO}}}} = k_{{\text{avg}}} - k_{{\text{CQD-CQD}}}$$

where *k*_avg_ is carrier transfer rate constant (*k*_ct_) of BTA@GO/PbS-PbX_2_ CQDs, *k*_CQD-CQD_ is carrier transfer rate constant between CQD-CQD, and *k*_CQD-BTA@GO_ is carrier transfer rate constant between CQD and BTA@GO. The calculated value of *k*_CQD-BTA@GO_ is 1.5 × 10^9^ s^−1^, and the percentage of carrier transfer between CQD-BTA@GO is about 27% (*k*_CQD-BTA@GO_/*k*_avg_). This means that about 27% of photogenerated holes can transport through BTA@GO channel in BTA@GO/PbS-PbX_2_ hybrid CQDs film. The larger carrier transfer rate of BTA@GO/PbS-PbX_2_ CQDs film will reduce the probability of charge carrier recombination which is confirmed by the EIS results (see Fig. 6b and Table S5) of solar cell devices. These results are beneficial for improving the *V*_OC_ and FF of CQDSCs device.

**Table 1:** Fitted time constants and the corresponding proportionality constants obtained from the TA decay curves of PbS-PbX_2_ CQDs film and BTA@GO/PbS-PbX_2_ hybrid CQDs film as shown in Fig. 3d

Sample	*A*_tr_	*τ*_tr_	*A*_ct_	*τ*_ct_	*A*_0_
PbS-OA	–	–	–	–	0.998 ± 0.002 (100%)
PbS-PbX_2_	− 0.041 ± 0.006 (4.0%)	21 ± 6 (ps)	− 0.758 ± 0.006 (74.6%)	243 ± 4 (ps)	− 0.217 ± 0.002 (21.4%)
BTA@GO/PbS-PbX_2_	− 0.033 ± 0.004 (3.4%)	23 ± 3 (ps)	− 0.802 ± 0.005 (80.0%)	180 ± 3 (ps)	− 0.167 ± 0.003 (16.6%)

**Fig. 4:**
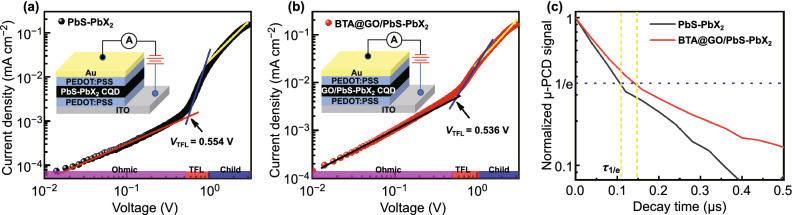
Dark *J*–*V* curves of hole-only devices based on PbS-PbX_2_ CQDs film **a** without and **b** with BTA@GO. **c** Normalized μ-PCD decay curves for the PbS-PbX_2_ CQDs film and BTA@GO/PbS-PbX_2_ hybrid CQDs film. The value of the blue short-dash line is 1/e, and the value of the yellow short-dash line is *τ*_1/e_ of carrier decay time, which are approximately 0.11 μs and 0.15 μs for the PbS-PbX_2_ CQDs film and BTA@GO/PbS-PbX_2_ hybrid CQDs film, respectively

Moreover, the carrier mobility and trap density of the CQDs film were also evaluated by using the space-charge-limited current (SCLC) method [35]. The structure and SEM image of the hole-only device (ITO/PEDOT:PSS/PbS CQDs/PEDOT:PSS/Au) are shown in Figs. 4 and S7, respectively. The typical dark current density–voltage (*J*–*V*) curves were obtained from SCLC measurement as shown in Fig. 4a, b, which can be divided into three regions: Child’s regime (*J*∝*V*^2^) at high bias voltage, Ohmic linear regime (*J*∝*V*) at the low bias voltage, and nonlinear trap-filled regime between them [36]. The carrier mobility (*μ*) in CQDs film can be calculated by fitting the *J*–*V* curve at Child’s regime with a Mott-Gurney law [37–40]:

2 $$J = \frac{9}{8}\varepsilon_{0} \varepsilon \mu \frac{{V^{2} }}{{L^{3} }}$$

where *J* is the current density, *ε*_0_ is the permittivity under vacuum, *ε* is the relative dielectric constant of PbS CQDs (about 14.5) [41], *V* is the applied voltage, and *L* is the thickness of PbS CQDs layer (about 330 nm). The calculated hole mobility of BTA@GO/PbS-PbX_2_ hybrid CQDs film is 0.0030 cm^2^ V^−1^ s^−1^, about 30% improvement as compared with hole mobility of 0.0023 cm^2^ V^−1^ s^−1^ for the PbS-PbX_2_ CQDs film. The introduction of BTA@GO can more effectively improve the hole mobility in the CQDs film. By the benefit from the additional BTA@GO charge transport channel, the BTA@GO/PbS-PbX_2_ hybrid CQDs film exhibits the higher carrier mobility than that of the control PbS-PbX_2_ CQDs film, which is consistent with the result of TA measurement. In addition, the trap-filled limit voltage (*V*_TFL_) can be extracted from the intersection of the curves between the trap-filled region and Ohmic linear region, which is determined by the trap density (*n*_trap_) in the device [42]:

3 $$V_{\text{TFL}} = \frac{{en_{\text{trap}} L^{2} }}{{2\varepsilon_{0} \varepsilon }}$$

The values of *V*_TFL_ for PbS-PbX_2_ CQDs film and BTA@GO/PbS-PbX_2_ hybrid CQDs film are 0.554 and 0.536 V, respectively. The calculated value of hole trap density for BTA@GO/PbS-PbX_2_ hybrid CQDs film is 7.89 × 10^15^ cm^−3^, which is lower than that of PbS-PbX_2_ CQDs solid film (8.16 × 10^15^ cm^−3^). This confirms that the bulk nano-heterojunction architectural for BTA@GO/PbS-PbX_2_ hybrid CQDs film can effectively passivate the trap state of PbS CQDs film [43], which is consistent with the result of TA measurement. Meanwhile, the free carrier lifetime (*τ*_1/e_) in the CQDs film obtained from microwave photoconductivity (μ-PCD) decay measurement is increased from 0.11 to 0.15  μs after BTA@GO introducing (Fig. 4c). The conductivity (*σ*) of the CQDs film is also measured (Fig. S8), and the evaluated *σ* values for PbS-PbX_2_ and BTA@GO/PbS-PbX_2_ CQD films are 5.18 × 10^−4^ and 6.16 × 10^−4^ S m^−1^, respectively. It indicates that there is some improvement in the carrier transport property of PbS-PbX_2_ CQD film after BTA@GO introducing. To put it from another angle, the BTA@GO-coupled CQDs film provides an effective carrier transport channel for enhanced carrier transport, which is beneficial for achieving higher *J*_SC_ and *V*_OC_ of CQDSCs.

### Photovoltaic Performance of BTA@GO/PbS-PbX_2_ Hybrid CQDs Film-Based Solar Cells

**Fig. 5:**
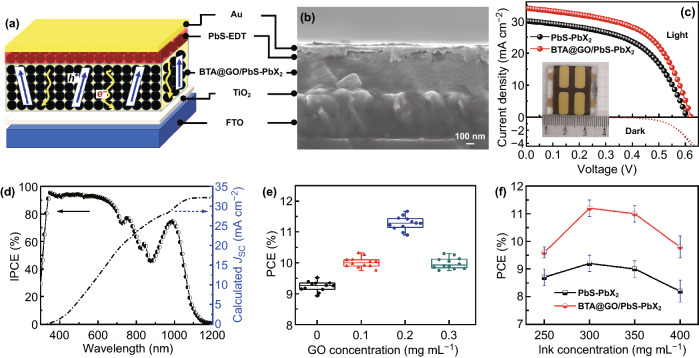
**a** Schematic diagram of the bulk nano-heterojunction structure CQDSCs with BTA@GO/PbS-PbX_2_ hybrid CQDs film. **b** Cross-sectional SEM image of the CQDSCs device. **c** *J*–*V* curves of CQDSCs based on the controlled PbS-PbX_2_ CQDs film and the BTA@GO/PbS-PbX_2_ hybrid CQDs film (the concentration of GO in CQDs ink is 0.2 mg mL^−1^). The inset is the photograph of CQDSCs device. **d** IPCE spectrum and integrated *J*_SC_ of BTA@GO/PbS-PbX_2_ CQDSCs. **e** Statistical distribution of PCEs for PbS-PbX_2_ CQDSCs devices with the different concentrations of GO in CQD inks from 0 to 0.3 mg mL^−1^. **f** Statistical distribution of PCEs for PbS-PbX_2_ CQDSCs devices which are fabricated by different concentrations of CQD inks from 250 to 400 mg mL^−1^

The efficient charge carrier transfer, enhanced carrier mobility and conductivity, as well as the extended free carrier lifetime in BTA@GO/PbS-PbX_2_ hybrid CQDs film are beneficial for thorough understanding of the photovoltaic performance of CQDSCs. A bulk nano-heterojunction device structure of FTO/TiO_2_/BTA@GO@PbS-PbX_2_/PbS-EDT/Au was designed and fabricated using BTA@GO/PbS-PbX_2_ hybrid CQDs ink as schematically illustrated in Fig. 5a, and the AFM images of PbS-PbX_2_ and BTA@GO/PbS-PbX_2_ CQD films and a cross-section SEM image of the device are presented in Figs. S9 and 5b, respectively. We fabricated and measured 12 devices for each type of the CQDSCs based on PbS-PbX_2_ CQDs film and BTA@GO/PbS-PbX_2_ hybrid CQDs film to ensure the repeatability of the hybrid inks and device fabrication. Figure 5c provides the *J*–*V* curves of the two type CQDSCs devices under one-sun (AM1.5G 100 mW cm^−2^) illumination. Table 2 shows the values of the performance parameters for those devices. The champion device (active area is 0.35 cm^2^) employing BTA@GO/PbS-PbX_2_ hybrid CQDs film achieved a PCE of 11.7%, with *J*_SC_, *V*_OC_, and FF of 33.9 mA cm^−2^, 0.622 V, and 55.5%, respectively. Additionally, there is no hysteresis in the device (Fig. S10 and Table S1). Compared to the control device which was fabricated with PbS-PbX_2_ CQDs film (PCE of 9.5%), all performance parameters of BTA@GO/PbS-PbX_2_ hybrid CQDs film-based CQDSCs were improved and the PCE was increased by 23%. Just as above mentioned, the charge transfer rate, carrier mobility and conductivity for the BTA@GO/PbS-PbX_2_ hybrid CQDs film are larger than those of the controlled PbS-PbX_2_ CQDs film. Thus, compared to PbS-PbX_2_ CQDs film-based device, the larger *J*_SC_ of BTA@GO/PbS-PbX_2_ hybrid CQDs film-based CQDSCs should be caused by the faster charge transfer rate, larger carrier mobility, and higher carrier extraction efficiency. The incident photon-to-current conversion efficiency (IPCE) spectrum and the calculated *J*_SC_ from the IPCE of the BTA@GO/PbS-PbX_2_ hybrid CQDs film-based CQDSCs are shown in Fig. 5d. The calculated *J*_SC_ (32.3 mA cm^−2^) is close to the value obtained from above *J*–*V* measurements. We optimized the adding amount of BTA@GO in the CQD inks for CQDSCs (Fig. 5e). The *J*_SC_, *V*_OC_, and FF of the devices firstly improve and then reduce with the optimized 0.2 mg mL^−1^ concentration of GO in the ink (Fig. S11 and Table S2). Excessive adding of BTA@GO may enhance the recombination in the device. We also optimized the thickness of CQDs layer by changing the concentration of CQD inks for CQDSCs (Fig. 5f). As shown in Fig. S12, with the increases in CQD inks concentration, the thickness of CQDs film increases. The *J*_SC_ of the both devices first grow sharply and then reduce slightly as the thickness increases, but sacrificing *V*_OC_ and FF (Fig. S12, Tables S3 and S4). It indicates that although thick CQDs layer can absorb more light (see Fig. S13) which benefits to generate charge carriers, the performance of the device is also constrained by the limit of carrier diffusion length and the increased trap density [16, 44].

**Table 2:** Performance parameters of CQDSCs based on PbS-PbX_2_ CQDs film and BTA@GO/PbS-PbX_2_ hybrid CQDs film. Light intensity for the measurement is one-sun

Devices	*J*_SC_ (mA cm^−2^)	*V*_OC_ (V)	FF (%)	PCE (%)
PbS-PbX_2_	30.1 ± 0.7 (30.1)	0.600 ± 0.008 (0.606)	51.3 ± 1.3 (52.3)	9.2 ± 0.3 (9.5)
BTA@GO/PbS-PbX_2_	33.2 ± 0.8 (33.9)	0.617 ± 0.007 (0.622)	54.5 ± 1.2 (55.5)	11.3 ± 0.4 (11.7)

The devices were measured under air at 20 °C with humidity of 45%. The values in parentheses are the parameters for the champion devices

**Fig. 6:**
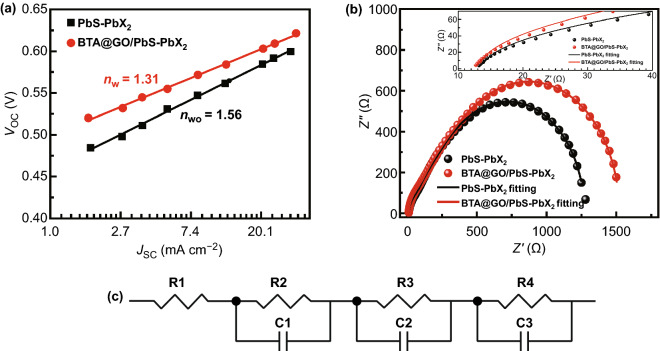
**a** *V*_OC_ plotted against the logarithm of *J*_SC_ in the CQDSCs device with and without BTA@GO. **b** Nyquist plots of PbS-PbX_2_ CQDs film and BTA@GO/PbS-PbX_2_ hybrid CQDs film-based CQDSCs in dark with 0.55 V applied bias, and the solid curves are the fitting results by using the equivalent circuit plotted in **c**. **c** Equivalent circuit of CQDSCs device

As we discussed above, the introduction of BTA@GO is beneficial for extracting holes from PbS CQD and provides a fast charge transport channel to carry the holes to the PbS-EDT hole-selective layer. This additional charge transport path and the bulk nano-heterojunction architecture are beneficial to reduce the probability of interfacial-assisted carrier recombination in CQDs layer which is caused by the trap states of PbS CQDs, and it is confirmed by the result of SCLC measurement (Fig. 4). The suppressed interfacial-assisted carrier recombination can weaken its contribution to *V*_OC_ deficit of the CQDSCs device. Diode ideality factor (*n*) is an important parameter of solar cells which can be used to reveal the carrier recombination process in the device. When the value of *n* approaches 1, it indicates that the carrier recombination process in solar cells device is mainly determined by charge carrier band-to-band directly recombination. On the contrary, when the value of *n* is much greater than unity (1 < *n* < 2), the interfacial-assisted recombination instead of the competition with band-to-band recombination mainly dominates the carrier recombination process in the device. We evaluate the diode ideality factor of the PbS-PbX_2_ CQDs film and BTA@GO/PbS-PbX_2_ hybrid CQDs film-based CQDSCs from a slope in *V*_OC_ plotted against the logarithm of the *J*_SC_ based on Eq. 4 [17]:

4 $$V_{\text{OC}} \propto \frac{{nk_{\text{B}} T}}{q}\ln \left( {J_{\text{SC}} } \right)$$

where *n*, *k*_B_, *T,* and *q* are the diode ideality factor, Boltzmann constant, temperature, and elementary charge, respectively. As shown in Fig. 6a, the estimated values of *n* are 1.56 and 1.31 for PbS-PbX_2_ CQDs film and BTA@GO/PbS-PbX_2_ hybrid CQDs film-based CQDSCs, respectively. The smaller *n* for BTA@GO/PbS-PbX_2_ hybrid CQDs film-based CQDSCs reflects the reduced interfacial-assisted recombination in the BTA@GO/PbS-PbX_2_ hybrid CQDs film layer comparing with the CQDs film without BTA@GO. It can be concluded from the above results that the introduction of BTA@GO into PbS-PbX_2_ CQDs film can significantly improve the carrier transport, diminish the recombination in CQDs film layer, and therefore enhance the *V*_OC_ and PCE.

So as to reveal the kinetic mechanism of the CQDSCs, EIS has been performed at negative applied bias. Figure 6b shows the Nyquist plots of CQDSCs based on PbS-PbX_2_ CQDs film and BTA@GO/PbS-PbX_2_ hybrid CQDs film under dark with 0.55 V applied bias. The series resistance (*R*_s_), recombination resistance (*R*_rec_), and chemical capacitance (*C*) of devices can be obtained (Table S5) by analyzing the EIS spectra with the equivalent circuits plotted in Fig. 6c. The fitted value of *R*_s_ for BTA@GO/PbS-PbX_2_ hybrid CQDs film-based device (12.3 Ω) is found to be smaller than that for PbS-PbX_2_ CQDs film-based device (12.9 Ω), this may be caused by the reduced resistance of the BTA@GO/PbS-PbX_2_ hybrid CQDs film. The *R*_rec_ of the device attributes to the sum of the low and intermediate frequency resistances. Compared with the PbS-PbX_2_ CQDs film-based CQDSCs (1165 Ω), a larger *R*_rec_ is obtained as 1411 Ω for BTA@GO/PbS-PbX_2_ hybrid CQDs film-based device. The value of carrier recombination rate constant (*k*_rec_) can be estimated by *k*_rec_=1/(*R*×*C*) and the corresponding *k*_rec_ values for the PbS-PbX_2_ CQDs film and BTA@GO/PbS-PbX_2_ hybrid CQDs film-based CQDSCs are 5.7×10^4^ and 3.9×10^4^ s^−1^, respectively. The smaller *R*_s_ as well as smaller *k*_rec_ values for BTA@GO/PbS-PbX_2_ hybrid CQDs film-based CQDSCs device indicate the improved carrier transport and reduced charge recombination in the device, which is favorable for improving the FF and *V*_OC_ of CQDSCs.

**Fig. 7:**
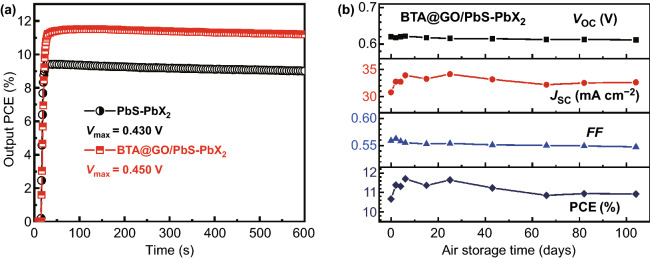
**a** Stable output test of PbS-PbX_2_ CQDs film and BTA@GO/PbS-PbX_2_ hybrid CQDs film-based CQDSCs at maximum output power condition which were measured under AM1.5 G 100 mW cm^−2^ light soaking in air. **b** Air storage stability of the BTA@GO/PbS-PbX_2_ hybrid CQDs film-based CQDSCs with indoor relative humidity of 40–65%

The stability is one of the important performance indicators of CQDSCs. The stable output curves of PCE of CQDSCs devices with and without BTA@GO as a function of time at the maximum output power condition were measured as shown in Fig. 7a. A stabilized output PCE of 11.5% at maximum power point (*J*_max_ of 25.6 mA cm^−2^ and *V*_max_ of 0.450 V) is achieved for the CQDSCs device based on BTA@GO/PbS-PbX_2_ hybrid CQDs film, which is higher and more stable than that of PbS-PbX_2_ CQDs film-based device (9.4%, at *J*_max_ of 21.9 mA cm^−2^ and *V*_max_ of 0.430 V). Thus, a small amount of BTA@GO adding can effectively improve the light-soaking stability of CQDSCs device. The long-term stability of BTA@GO/PbS-PbX_2_ hybrid CQDs film-based CQDSCs was also investigated. The device was stored and tested in air with the humidity of 40–65%. As shown in Fig. 7b, the BTA@GO/PbS-PbX_2_ hybrid CQDs film-based CQDSC exhibits good air storage stability for over 105 days, and the photovoltaic performance of the device was increased at the first 6 days. With storage time going on, the *V*_OC_ and FF of the device are slightly reduced which may be caused by the desorption of EDT from PbS CQDs surface in PbS-EDT layer in turn changed the energy level and trap density of PbS-EDT layer [45, 46]. Finally, the PCE of BTA@GO/PbS-PbX_2_ hybrid CQDs film-based CQDSC retained about 93% of its peak value after 105 days, from 11.7 to 10.9%.

## Conclusions

In summary, we artfully use the BTA-modified GO in the BTA@GO/PbS-PbX_2_ hybrid CQDs ink to build a bulk nano-heterojunction architecture CQDSCs. The introduction of BTA@GO provides effective carrier transport channel which improves the charge transfer rate, carrier mobility, and conductivity, as well as the carrier lifetime of PbS-PbX_2_ CQDs film. The bulk nano-heterojunction architecture can reduce the carrier recombination rate and improve the carrier extraction in the device. Combined those advantages, all of the performance parameters of CQDSCs are enhanced. A stable output and air storage relatively large active area (0.35 cm^2^) BTA@GO/PbS-PbX_2_ hybrid CQDs film-based CQDSC with a champion PCE of 11.7% was obtained, and the PCE shows a marked increase (23.1%) compared with the PbS-PbX_2_ CQD-based CQDSCs. Our results suggest that improving the hole mobility of CQDs active layer and the bulk nano-heterojunction architecture is promising to further improve the performance of CQDSCs in future.

## Electronic Supplementary Material

Below is the link to the electronic supplementary material.

Supplementary material 1 (DOCX 3774 docx)
