# Supplementary material for: Surface-Modified Graphene Oxide/Lead Sulfide Hybrid Film-Forming Ink for High-Efficiency Bulk Nano-Heterojunction Colloidal Quantum Dot Solar Cells
Source: Nanomicro Lett. 2020 May 16;12:111. doi: 10.1007/s40820-020-00448-8 (PMC7770832; doi:10.1007/s40820-020-00448-8)
Supplement: Supplementary file 1 — Supplementary material 1 (DOCX 3774 docx) [file 40820_2020_448_MOESM1_ESM.docx]

**Supporting information**

Surface-modified graphene oxide/lead sulfide hybrid film-forming ink for high efficiency bulk nano-heterojunction colloidal quantum dot solar cells

Yaohong Zhang ^1^, Guohua Wu ^2,*^, Chao Ding ^1^, Feng Liu ^1^, Dong Liu ^1^, Taizo Masuda ^3^, Kenji Yoshino ^4^, Shuzi Hayase ^1^, Ruixiang Wang ^5,*^, Qing Shen ^1,*^

^1^ Faculty of Informatics and Engineering, The University of Electro-Communications, Tokyo 182-8585, Japan.

^2^ School of Materials Science & Engineering, Shaanxi Normal University, Xi’an 710119, China.

^3^ X-Frontier Division, Toyota Motor Corporation, Shizuoka, 471-8571, Japan

^4^ Department of Electrical and Electronic Engineering, Miyazaki University, Miyazaki 889-2192, Japan

^5^ Beijing Engineering Research Centre of Sustainable Energy and Buildings, Beijing University of Civil Engineering and Architecture, Beijing 102616, China.

Corresponding Author

* G. Wu. E-mail: [ghwu@snnu.edu.cn](mailto:ghwu@snnu.edu.cn)

* R. Wang. E-mail: wangruixiang@bucea.edu.cn

* Q. Shen. E-mail: shen@pc.uec.ac.jp


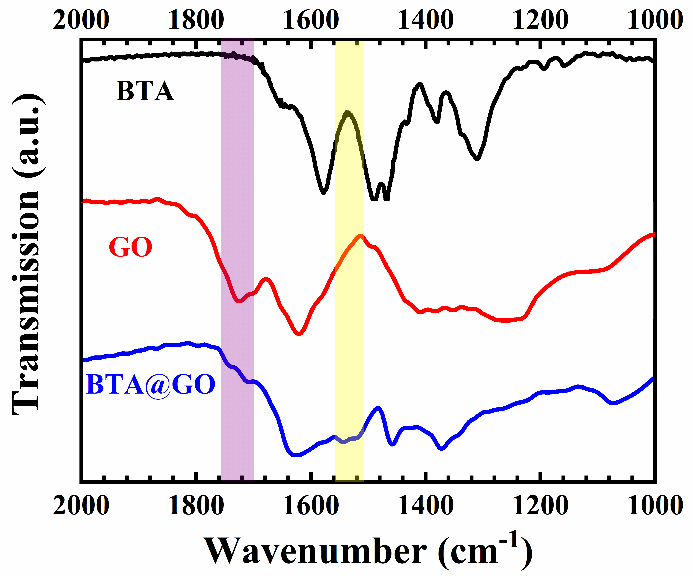


**Fig. S1** FT-IR spectra of BTA, GO and GO@BTA.


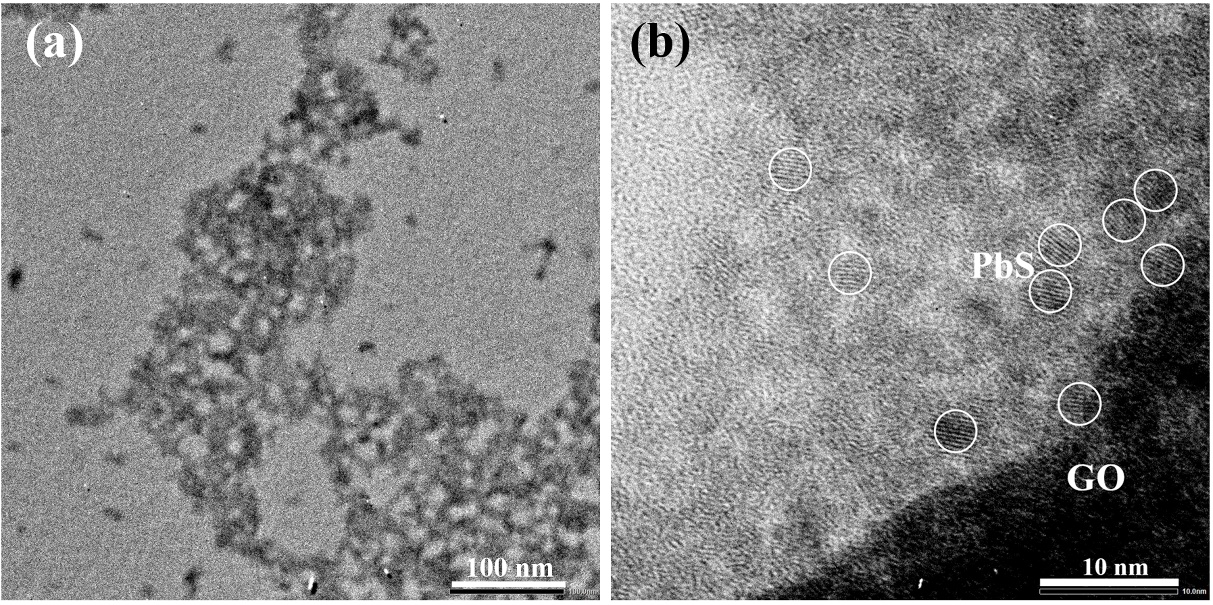


**Fig. S2** (a) TEM image of PbS-PbX_2_ CQDs and (b) HR-TEM image of PbS CQDs coupled with BTA@GO.


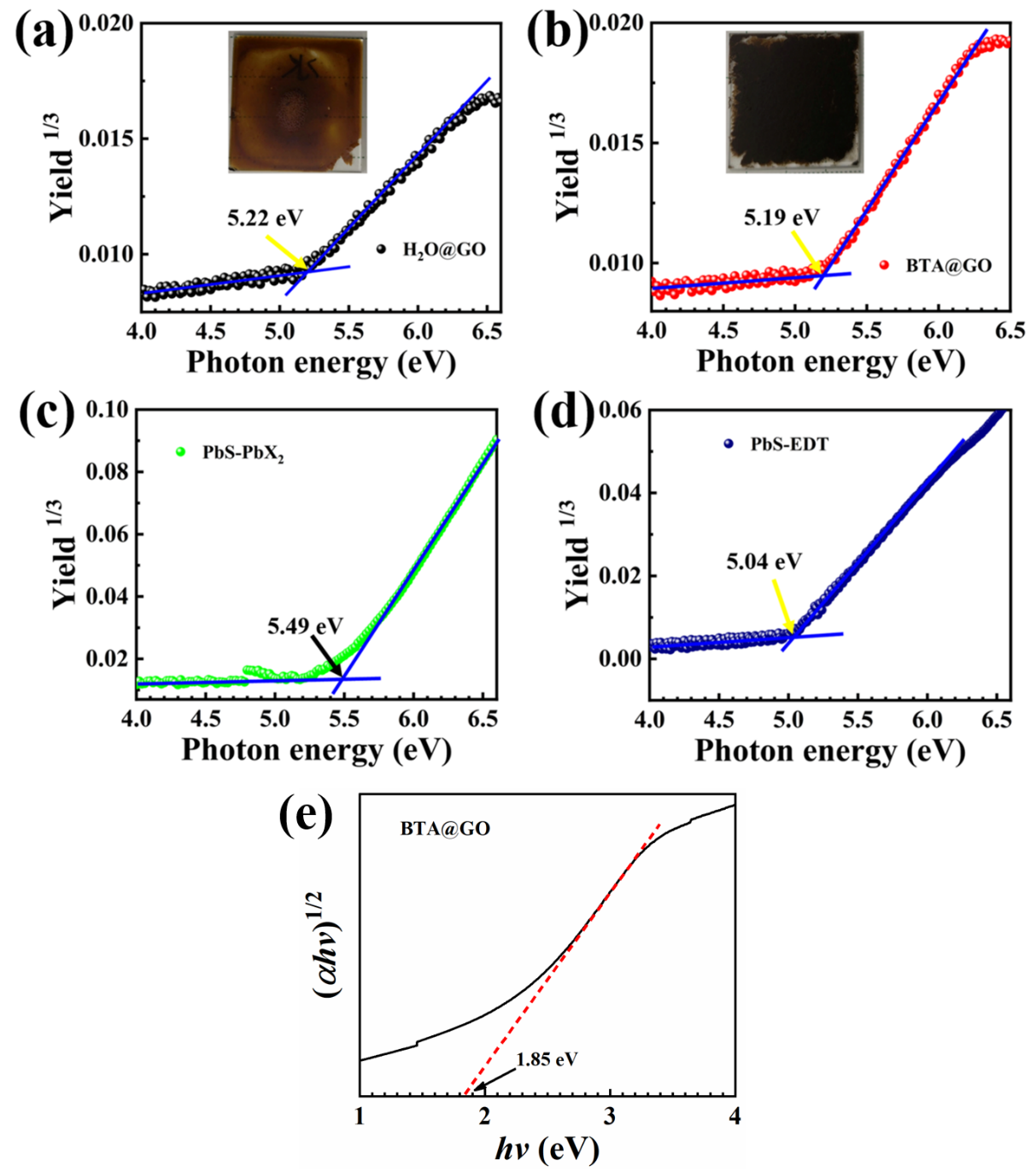


**Fig. S3** PYS spectra of (a) H_2_O@GO, (b) BTA@GO, (c) PbS-PbX_2_ CQDs film, (d) PbS-EDT CQDs film, and (e) Tauc plot of (*αhν*)^1/2^ against the photon energy (*hν*) for BTA@GO, respectively. The inset pictures in (a) and (b) are the photographs of H_2_O@GO and BTA@GO films, respectively.

GO is a heavily oxygenated monolayer material consisting of a variety of functional groups which has both sp2 and sp3 clusters and sp2 clusters mostly embedded inside sp3 cluster, thus the bandgap of GO like disorder structure. The optical bandgap estimated from Tauc plot considering an indirect bandgap is 1.85 eV for BTA@GO as shown in Fig. S3e.[1-2]


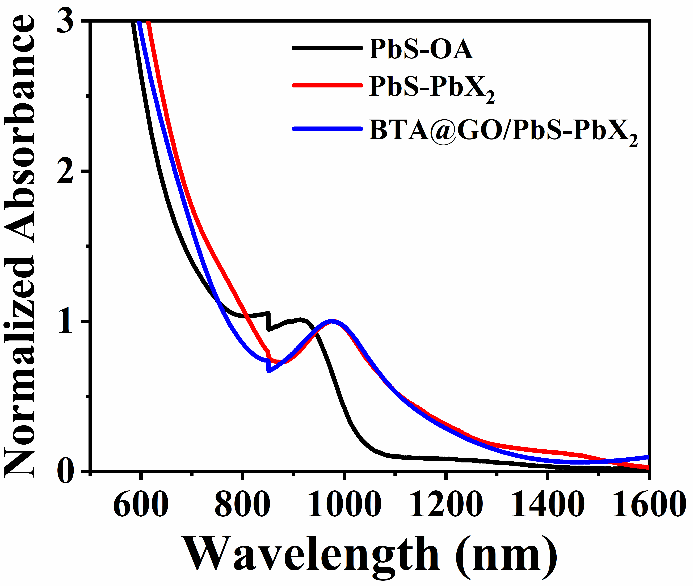


**Fig. S4** Normalized absorption spectra of TA film samples.


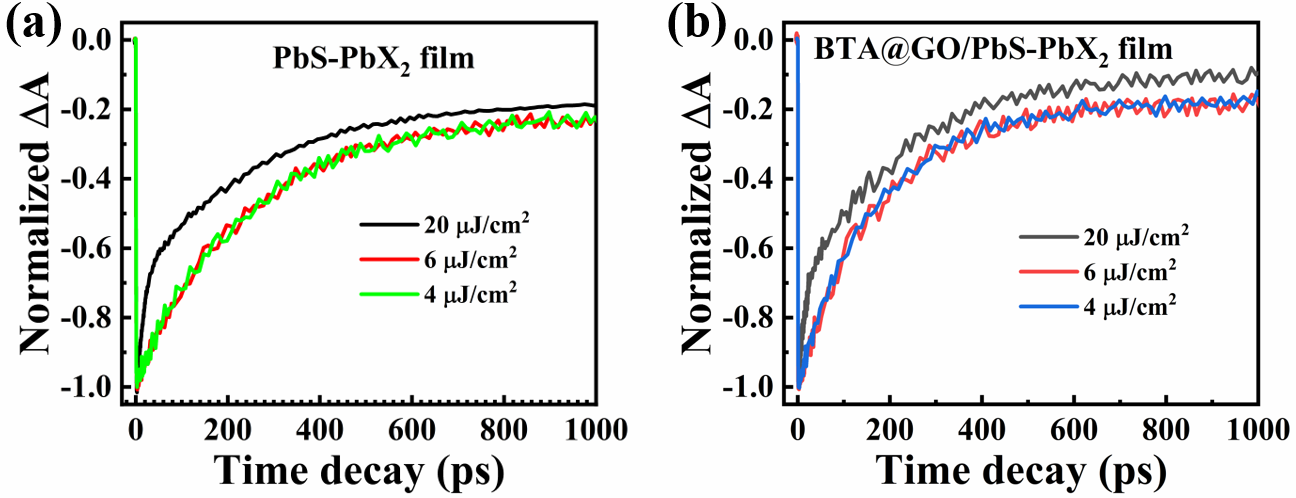


**Fig. S5** Power dependent TA decay spectra of (a) PbSe-PbX_2_ CQDs film and (b) BTA@GO/PbSe-PbX_2_ hybrid CQDs film. The sample is pumped by 470 nm and probed at 970 nm. When the pump fluence reduced to 6 μJ/cm^2^, the signal of Auger recombination disappeared.


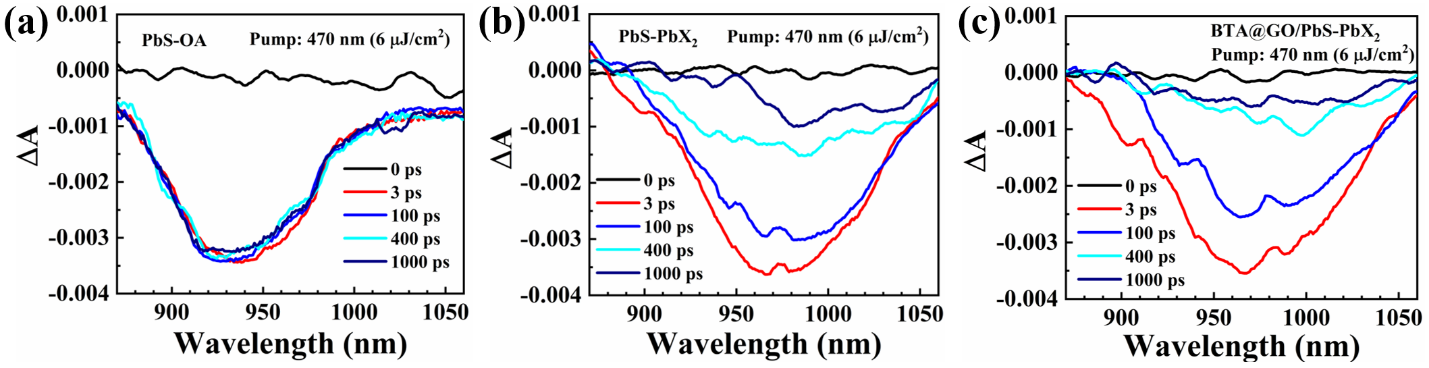


**Fig. S6** TA spectra for (a) PbS-OA, (b) PbS-PbX_2_ and (c) BTA@GO/PbS-PbX_2_ CQD films at 0, 3, 100, 400, and 1000 ps. The pumped wavelength is 470 nm with a pulse fluence of 6 μJ/cm^2^.


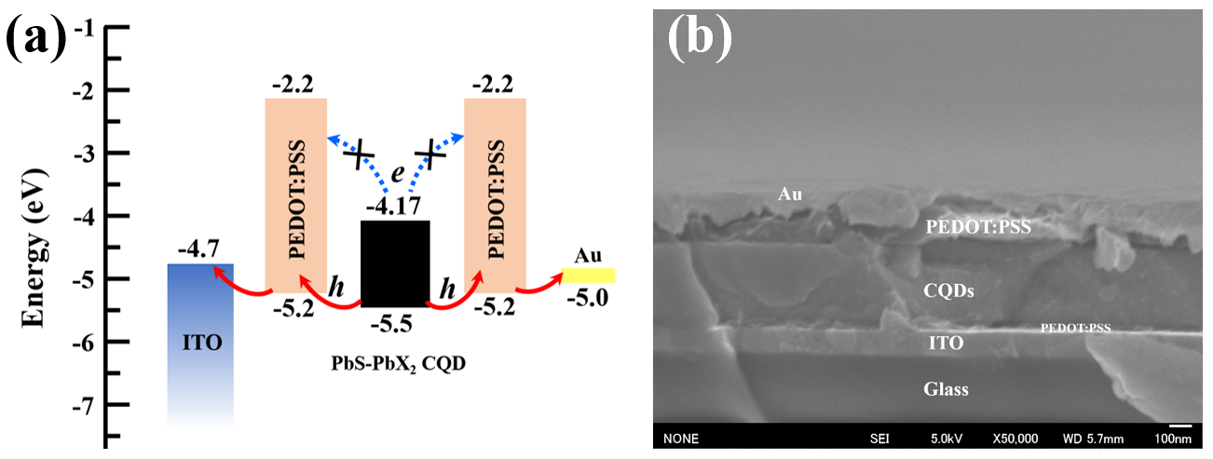


**Fig. S7** (a) Schematic energy band diagrams of ITO, PEDOT:PSS, PbS-PbX_2_ CQD and Au. (b) SEM cross section image of hole-only device.

The hole-only device with a structure of ITO/PEDOT/CQD/PEDOT/Au was fabricated by spin-coating a mixture of PEDOT:PSS solution and methanol with a volume ratio of 1:3 on ITO at 4000 r.p.m. After annealing ITO/PEDOT substrate at 150°C for 15 min, the CQD ink was deposited on PEDOT:PSS layer by spin-coating. Finally, a thick PEDOT:PSS layer was deposited on CQD layer by spin-coating PEDOT:PSS solution/methanol mixture solution with a volume ratio of 1:1 followed by annealing in N_2_ atmosphere.


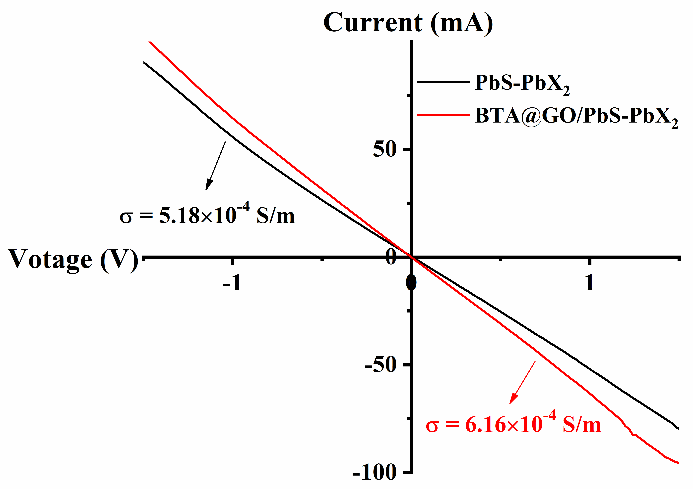


**Fig. S8** I-V curves of Glass/Au/CQD film/Au devices based on PbS-PbX_2_ and BTA@GO/PbS-PbX_2_ CQD films which were measured under dark condition.

The values of resistance (*R*) fitted from the I-V curves are 18.2 Ω and 15.3 Ω for PbS-PbX_2_ and BTA@GO/PbS-PbX_2_ CQD films, respectively. The conductivity (σ) of the film can be calculated from following equation:

$$\sigma=\frac{d}{R\times S} (S1)$$

where *d* is the thickness of the film (about 330 nm) and *S* is the area of the device (0.35 cm^2^). The evaluated σ values for PbS-PbX_2_ and BTA@GO/PbS-PbX_2_ CQD films are 5.18×10^-4^ S/m and 6.16×10^-4^ S/m, respectively.


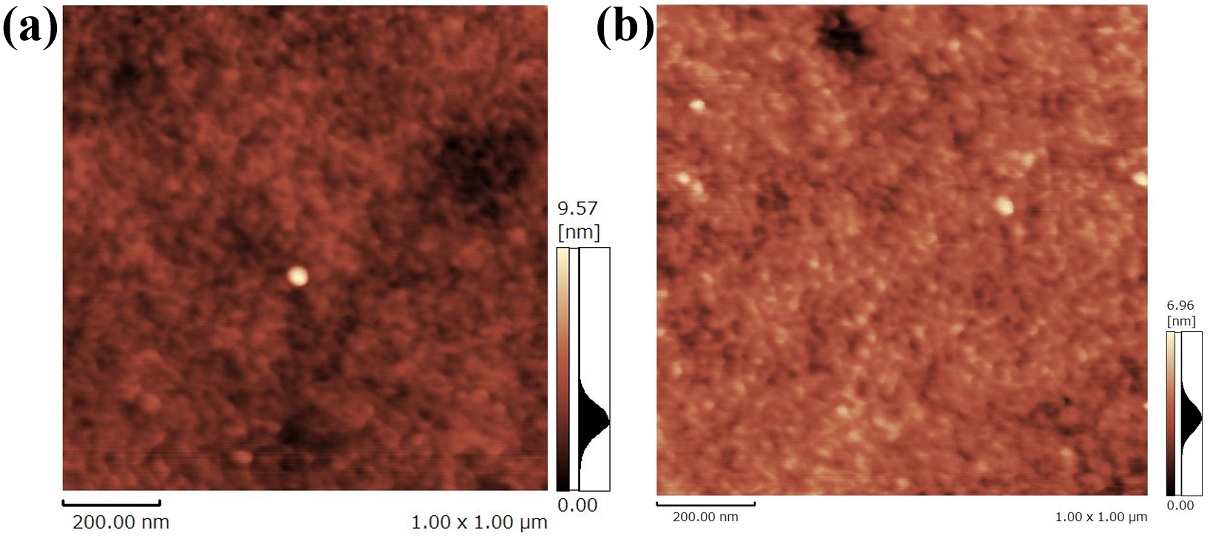


**Fig. S9** AFM images of (a) PbS-PbX_2_ and (b) BTA@GO/PbS-PbX_2_ CQD films.


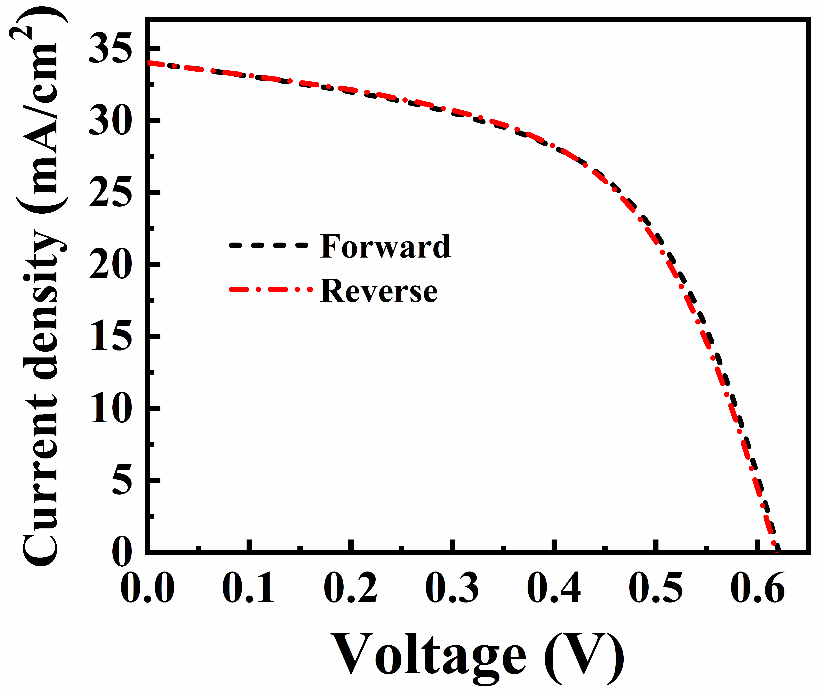


**Fig. S10** *J-V* curves of BTA@GO/PbS-PbX_2_ hybrid CQDs film (GO concentration is 0.2 mg/mL, CQD ink concentration is 300 mg/mL) based CQDSCs measured by forward (short circuit → open circuit) and reverse (open circuit → short circuit) scans with 0.01 V voltage steps and 200 ms delay times under AM 1.5G 100 mW/cm^2^ illumination.

**Table S1** Performance details of BTA@GO/PbS-PbX_2_ hybrid CQDs film based CQDSCs as shown in Fig. S10.

|  | *J*_SC_ (mA/cm^2^) | *V*_OC_ (V) | *FF* (%) | PCE (%) |
| --- | --- | --- | --- | --- |
| Forward Scan | 33.9 | 0.620 | 55.4 | 11.6 |
| Reverse Scan | 34.0 | 0.618 | 55.2 | 11.6 |
| Average | 34.0 | 0.619 | 55.3 | 11.6 |


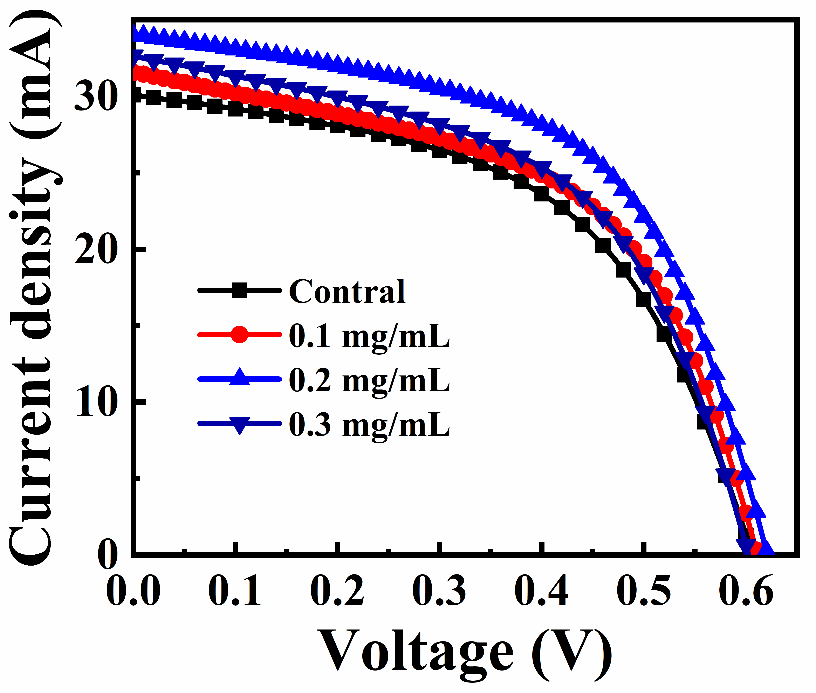


**Fig. S11** J–V curves of the CQDSCs devices with the different concentrations of GO from 0 mg/mL to 0.3 mg/mL (CQD ink concentration is 300 mg/mL).

**Table S2** Performance details of the PbS-PbX_2_ CQD inks based devices which are fabricated by using different GO concentration inks as shown in Fig. S11.

| GO concentration (mg/mL) | *J*_sc_ (mA/cm^2^) | *V*_oc_ (V) | *FF* (%) | PCE (%) |
| --- | --- | --- | --- | --- |
| 0 | 30.1 | 0.606 | 52.3 | 9.5 |
| 0.1 | 31.5 | 0.611 | 53.2 | 10.3 |
| 0.2 | 33.9 | 0.621 | 55.4 | 11.7 |
| 0.3 | 32.6 | 0.602 | 52.4 | 10.3 |


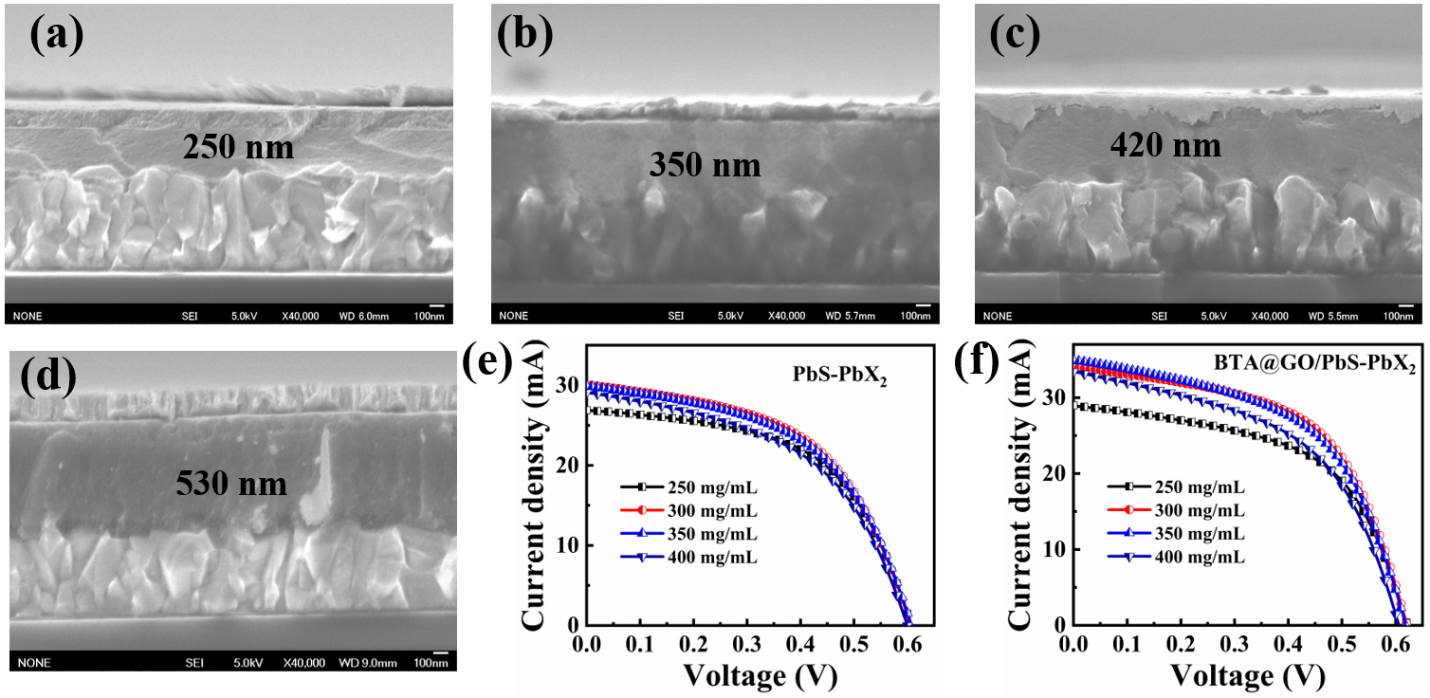


**Fig. S12** SEM cross section images of CQDSCs with different BTA@GO/PbS-PbX_2_ hybrid CQDs layer thicknesses (a, 250 mg/mL ink; b, 300 mg/mL ink; c, 350 mg/mL ink; d, 400 mg/mL ink), and *J-V* curves of the devices (e, without GO; f, with GO (0.2 mg/mL)).

**Table S3**. Performance details of the PbS-PbX_2_ CQD ink based devices which are fabricated by using different CQD concentration inks as shown in Fig. S12e.

| Ink concentration (mg/mL) | *J*_sc_ (mA/cm^2^) | *V*_oc_ (V) | *FF* (%) | PCE (%) |
| --- | --- | --- | --- | --- |
| 250 | 26.9 | 0.607 | 54.8 | 9.0 |
| 300 | 30.1 | 0.606 | 52.3 | 9.5 |
| 350 | 29.9 | 0.604 | 51.6 | 9.3 |
| 400 | 29.1 | 0.600 | 49.4 | 8.6 |

**Table S4** Performance details of the BTA@GO/PbS-PbX_2_ hybrid CQD inks based devices which are fabricated by using different CQD inks as shown in Fig. S12f.

| Ink concentration (mg/mL) | *J*_sc_ (mA/cm^2^) | *V*_oc_ (V) | *FF* (%) | PCE (%) |
| --- | --- | --- | --- | --- |
| 250 | 29.0 | 0.621 | 54.9 | 9.9 |
| 300 | 34.0 | 0.621 | 55.4 | 11.7 |
| 350 | 34.9 | 0.619 | 52.5 | 11.4 |
| 400 | 33.4 | 0.606 | 50.5 | 10.2 |





**Fig. S13** The absorption spectra of BTA@GO/PbS-PbX_2_ hybrid CQD inks based devices which are fabricated by using different CQD inks.

**Table S5** Fitted resistances and capacitances from the EIS spectra of PbS-PbX_2_ CQDs and BTA@GO/PbS-PbX_2_ hybrid CQDs based devices as shown in Fig. 6b.

| **Device** | ***R*_s_ (Ω)** | ***R*_low_ (Ω)** | ***R*_inter_ (Ω)** | ***C*_low_ (F)** | ***C*_inter_ (F)** | |
| --- | --- | --- | --- | --- | --- | --- |
| PbS-PbX_2_ | 12.9 | 1035 | 130 | 1.13E-7 | 1.34E-7 | |
| BTA@GO/PbS-PbX_2_ | 12.3 | 1215 | 196 | 1.1E-7 | 1.3E-7 |  |

The value of the *R*_rec_ was calculated by using follow equation:

$$R_{rec}=R_{low}+R_{inter} (S2)$$

where *R*_low_ and *R*_inter_ are resistances at low and intermediate frequency, respectively. The values of *R*_rec_ are 1165 Ω and 1411 Ω for PbS-PbX_2_ film and BTA@GO/PbS-PbX_2_ hybrid CQDs film based devices, respectively.

The value of *k*_rec_ was calculated by using follow equation:

$$k_{rec-low}=\frac{1}{R_{low}\times C_{low}} (S3)$$

$$k_{rec-inter}=\frac{1}{R_{inter}\times C_{inter}} (S4)$$

where *C*_low_ and *C*_inter_ are capacitances at low and intermediate frequency, respectively. The values of *k*_rec-low_ are 8.5×10^3^ s^-1^ and 7.5×10^3^ s^-1^ for PbS-PbX_2_ film and BTA@GO/PbS-PbX_2_ hybrid CQDs film based devices, respectively. And the values of *k*_rec-inter_ for PbS-PbX_2_ film and BTA@GO/PbS-PbX_2_ hybrid CQDs film based devices are 5.7×10^4^ s^-1^ and 3.9×10^4^ s^-1^, respectively. In the solar cells device, the recombination rate mainly dependents on the fast recombination process. Thus, the carrier recombination rates for PbS-PbX_2_ film and BTA@GO/PbS-PbX_2_ hybrid CQDs film based devices can be approximated as 5.7×10^4^ s^-1^ and 3.9×10^4^ s^-1^, respectively.

**Reference**

1. T.F. Yeh, F.F. Chan, C.T. Hsieh, H. Teng. Graphite Oxide with Different Oxygenated Levels for Hydrogen and Oxygen Production from Water under Illumination: The Band Positions of Graphite Oxide. J. Phys. Chem. C, **115**(45), 22587-22597 (2011). https://doi.org/10.1021/jp204856c

2. M.A. Velasco-Soto, S.A. Pérez-García, J. Alvarez-Quintana, Y. Cao, L. Nyborg, L. Licea-Jiménez. Selective Band Gap Manipulation of Graphene Oxide by Its Reduction with Mild Reagents. Carbon, **93**, 967-973 (2015). https://doi.org/10.1016/j.carbon.2015.06.013
